# Recipes for Health: A Community-Based Nutrition and Culinary Intervention

**DOI:** 10.7759/cureus.32322

**Published:** 2022-12-08

**Authors:** Sylvia Lillquist, Gabriela Ruiz Barnecett, Natalie Flexman, Nadine Mikati

**Affiliations:** 1 Nutrition, Nova Southeastern University Dr. Kiran C. Patel College of Osteopathic Medicine, Davie, USA; 2 Osteopathic Medicine, Nova Southeastern University Dr. Kiran C. Patel College of Osteopathic Medicine, Davie, USA

**Keywords:** mindful eating, weight loss, eating behaviors, dietary life change, dietary guidelines, virtual education, nutrition and food science, nutrition and metabolism, diabetes and nutrition, nutrition

## Abstract

Background: Obesity is a major public health concern in the United States, especially since it has been associated with an increased incidence of multiple co-morbidities. Positive eating behavior modifications learned through nutrition education sessions are the main interventions proposed to target overweight and obesity.

Objective: The aim of this study was to determine if nutrition education and hands-on cooking classes will result in improvement in eating habits and cooking skills to manage chronic disease.

Methods: A convenience sample of 21 participants were recruited from primary health clinics in Miami-Dade, Broward, and Palm Beach counties. A total of eight weekly virtual lessons were conducted, which included both a culinary and a nutrition education portion. At baseline and post-intervention, participants filled out a validated questionnaire with questions related to nutrition knowledge and behavior, fruit and vegetable consumption, and cooking skills. Weight was self-reported. Statistical analysis was conducted using IBM SPSS Statistics for Windows, Version 27.0 (Released 2020; IBM Corp., Armonk, New York, United States) and included descriptive statistics and a paired t-test to compare pre- and post-intervention data.

Results: Throughout the eight weeks, subject attendance ranged from 61-95%. Nineteen participants completed the post-intervention questionnaire. Results showed a statistically significant mean weight loss of 3.74 ±5.26 lbs (p=0.006) and a statistically significant mean BMI change of -0.66 ±0.86 (p=0.004) at post-intervention compared to baseline. In addition, subjects reported increased confidence in dietary habits and culinary skills post-intervention.

Conclusion:* *Our results show exciting data in support of this project’s objectives that a healthy cooking intervention can increase nutrition knowledge, increase confidence in healthy food choices, increase confidence in food preparation skills, and improve weight and BMI in participants.

## Introduction

Obesity is a rampant and growing issue in South Florida and the United States. As of 2019, 27% of adults in Florida were obese, whereas, 37.6% of Floridian adults were overweight [1]. Research shows that obesity is known for increasing the risk for chronic diseases such as cardiovascular disease, type 2 diabetes, renal disease, and cancer [2-3]. The comorbidities have been shown to have a detrimental impact on quality of life [4], and affect an individual’s sleep, relationships, work-life, and mental health status. Even more concerning is the fact that within the next 10 years, the prevalence of obesity has been predicted to increase to 40% worldwide [2]. Obesity has been found more prevalent in individuals with lower educational attainment, low income, food insecurity/accessibility, low nutrition literacy, and lack of practical meal preparation skills.

Additionally, dietary intake is also an important contributing factor given that it is influenced by environmental variables including food availability, variety, energy density, and portion sizes [5]. It has been found that the incidence of obesity has risen in parallel with larger portion sizes that are being served in restaurants, and ready-to-eat foods [6]. Lack of cooking skills and nutrition knowledge translates to more foods being eaten outside of the home at places such as restaurants, convenience stores, and fast-food restaurants. These foods have been shown to be higher in sodium, fat, and calories, which contribute to a higher BMI and its existing comorbidities. In contrast, meals prepared within the home are more likely to be lower in calories, fat, sugar, and portion sizes. Thus, cooking at home is a healthy solution that can increase diet quality and promote healthy food intake, which can help reduce the prevalence of diet-related chronic diseases [7].

Even with the previously stated knowledge, individuals often struggle to adjust their cooking and eating habits since they are not equipped with proper nutrition or culinary education [5-7]. Therefore, in this study, we compared nutrition knowledge, eating habits, cooking skills, and weight in patients before and after an eight-week culinary and nutrition intervention. This curriculum was infused with cooking demonstrations to provide individuals with the necessary tools, knowledge, and skills to change their dietary habits.

This article was previously presented as a poster at the 2022 Food and Nutrition Conference & Exposition (FNCE) hosted by the Academy of Nutrition and Dietetics on October 11, 2022, in Orange County.

## Materials and methods

Intervention

This research project was an intervention study with a pre/post design. The study consisted of eight virtual nutrition/cooking lessons conducted via the Zoom platform (Zoom Video Communications, Inc., San Jose, California, United States), where participants would meet once a week. Each virtual lesson lasted between 90-120 minutes. Prior to the classes beginning, participants were emailed a printable workbook that included a weekly grocery list, recipe instructions, nutrition facts, a meal planning guide, food safety tips, and a knife skills guide. The first part of the class was focused on nutrition education led by graduate nutrition students and registered dietitians while the second portion was facilitated by the Common Threads chef instructor (Common Threads, Austin, Texas, United States). The recipes provided to participants were based on the “Recipes For Health” curriculum provided by Common Threads and Baptist Health (Baptist Healthcare System, Louisville, Kentucky, United States) and further revised by graduate nutrition students to ensure that the content matched the most recent dietary guidelines. Topics for the sessions consisted of weight loss, mindful eating, and relaying facts to participants on carbohydrates, fats, sodium, and their relation to chronic disease (Table 1).

**Table 1:** Topics that were covered throughout the course of the eight weeks

Weeks	Topic Covered
Week 1	Fad or Facts (Weight Management)
Week 2	Meal Planning and Healthy Weight
Week 3	Nutrition Labels
Week 4	Mind Your Habits
Week 5	Nutrition and Fats
Week 6	Nutrition and Blood Pressure
Week 7	Truth About Carbs
Week 8	Diabetes Myths

**Figure 1:**
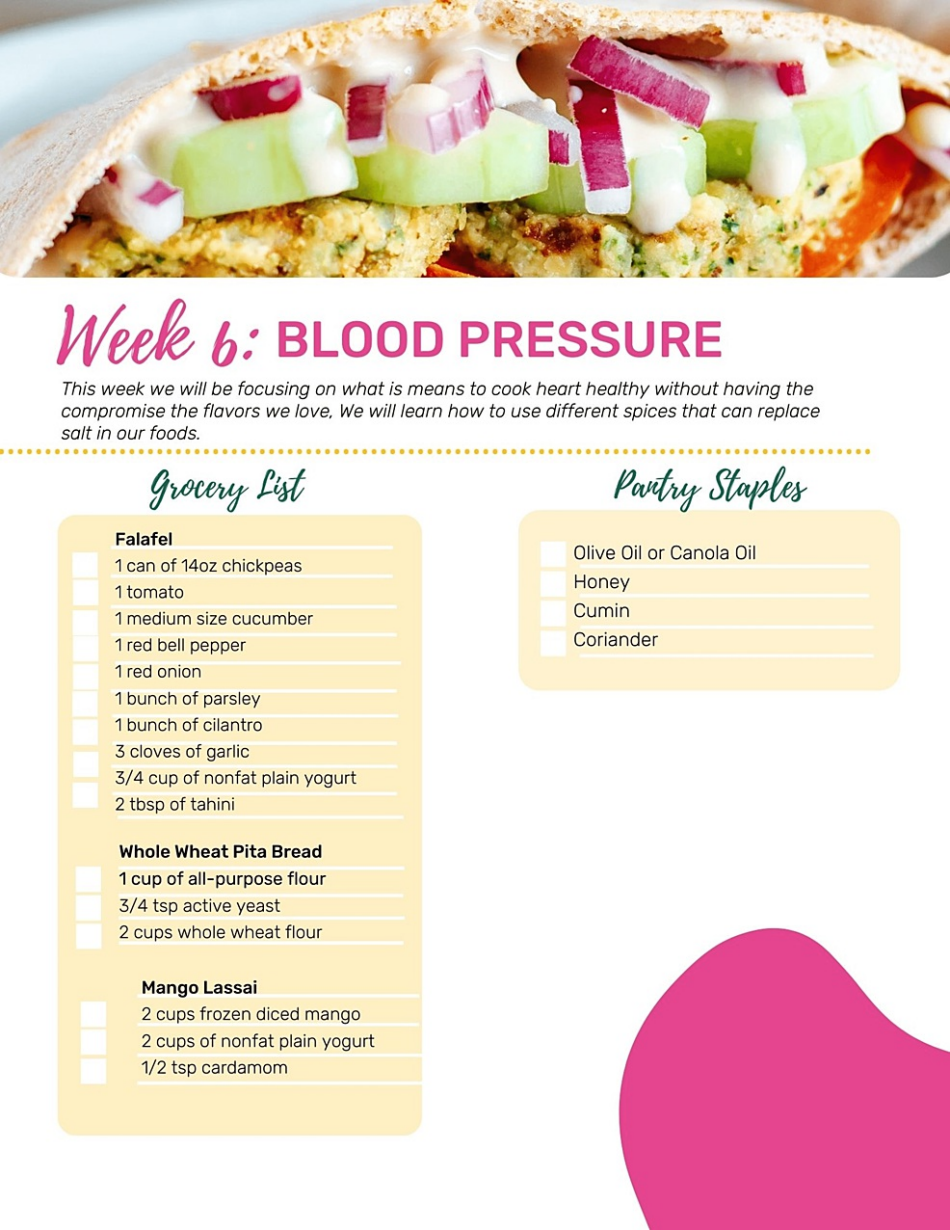
Week six meal plan Source: Used with permission from Common Threads, Austin, Texas, United States

**Figure 2:**
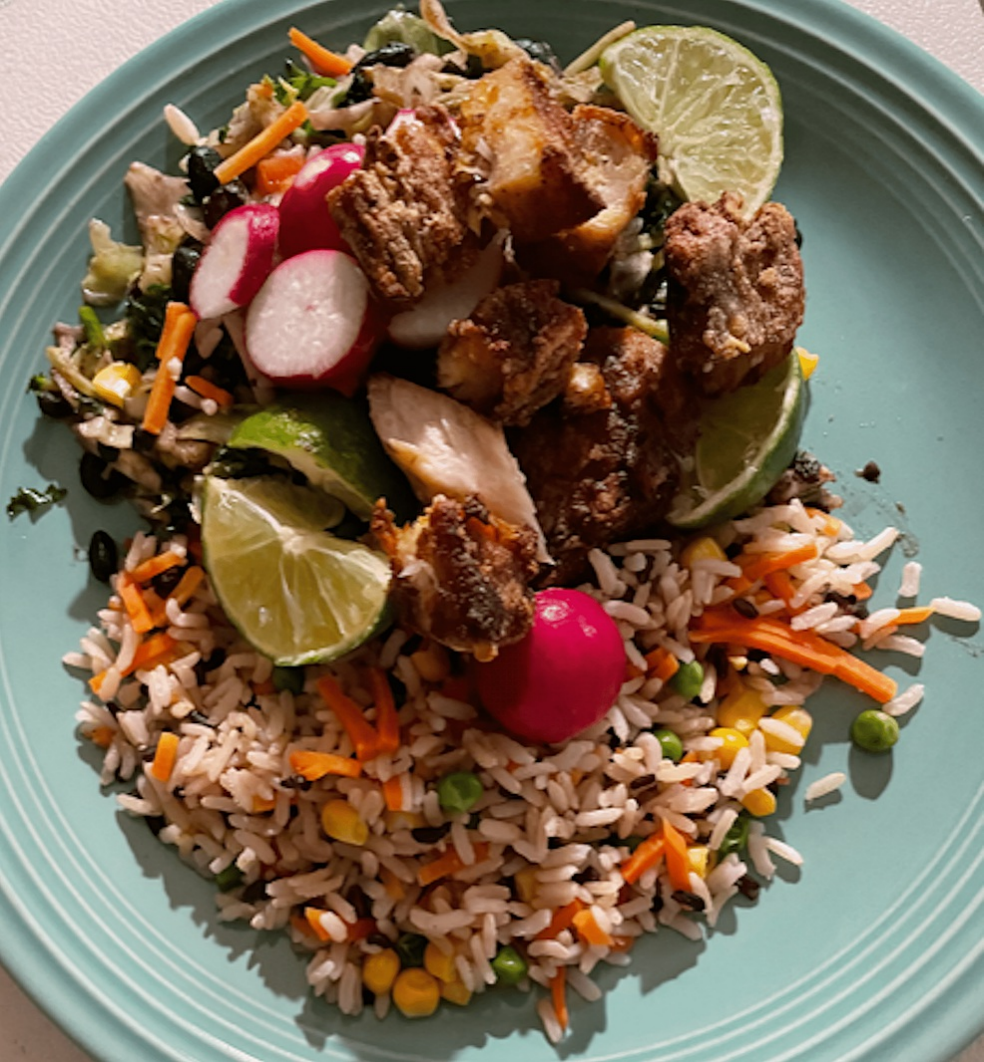
Sample participant photo from week eight of the intervention. Participants made a healthy version of a Chinese Stir-Fry.

The participants received a curriculum workbook that included the recipes for each week, along with cooking and meal-planning tips (Figure 1). At the end of the session, participants would send photographs of the recipes they had cooked (Figure 2). 

Recruitment

A convenience sample was recruited from local health clinics across the tri-county area in South Florida. To be included in the study, participants had to meet the following criteria: (1) reside in Miami-Dade, Broward, or Palm Beach counties, (2) be over the age of 18, (3) be proficient in English, (4) have access to a computer/smartphone, (5) have access to a full kitchen, and (6) have an existing chronic disease such as pre-diabetes, diabetes, heart disease, or overweight/obese (BMI > 25). Exclusion criteria included recent surgery within six months, taking medications that can interfere with body weight, physical or mental disability, autism, or cancer or HIV diagnosis. Participants that met the inclusion criteria were screened and consented. A cap was set at 21 participants due to grant budget availability. Institutional Review Board approval was obtained from Nova Southeastern University prior to the commencement of the study (approval number: 2020-605-NSU). All participants received a $15 gift card for grocery reimbursement after each session attended.

Data collection

REDCap (Vanderbilt University, Nashville, Tennessee, United States) was used to collect pre- and post-intervention data. Participants were given a demographic questionnaire at baseline through which information about marital status, annual income, gender, ethnicity, and other factors were obtained. Following that, participants were provided with a shortened version (38 questions) of the validated questionnaire by Condrasky and colleagues [8] to collect data on nutrition knowledge and behaviors as well as food preparation skills. This validated questionnaire was given pre- and post-intervention to determine if any attitudes or behaviors changed among the participants. In addition to questionnaires, participants were asked to self-report their weight at baseline and post-intervention to determine any weight and BMI changes.

Data analysis

To analyze the data, we used descriptive statistics to obtain the mean. A principal component factor analysis was conducted to reduce the number of dimensions in the data collected. This step created two sets of variables, Enjoyment and Health, and Confidence. Those factors were then subjected to paired sample t-tests to compare pre- and post-intervention data, specifically related to weight and BMI changes. IBM SPSS Statistics for Windows, Version 27.0 (Released 2020; IBM Corp., Armonk, New York, United States) was used and statistical significance was set at p<0.05. First, participants were asked about their confidence in different areas such as cooking, planning nutritious meals, and eating the recommended amounts of fruits and vegetables. They were given the option of 1 (not confident at all), 2 (not very confident), 3 (neither confident nor not confident), 4 (confident), or 5 (extremely confident). Next, they were asked a variety of questions such as if they enjoy cooking and if cooking is a good use of their time. Participants answered 1 (strongly disagree), 2 (disagree), 3 (neither agree nor disagree), 4 (agree), or 5 (strongly agree). Participants were also asked if they have fresh fruit/vegetables in their refrigerator. The answer options were yes or no. 

## Results

Characteristics of the sample

Of the 21 participants who responded to the survey, 85.7% (n=18) were women and 14.3% (n=3) were men. While the majority of the participants (38.1%, n=8) were over the age of 56, there was versatility in the other groups where 14.3% (n=3) of participants were in the age group of 18-25 years old, 14.3% (n=3) were in the age group of 26-35 years, 19% (n=4) were in the age group of 36-45 years, and 14.3% (n=3) were in the age group of 46-55 years. Regarding ethnicity, 57.1% (n=12) identified as Hispanic or Latino, 23.8% (n=5) were White, 14.3% (n=3) were Black or African American, and 4.8% (n=1) were Asian or Pacific Islander. For employment status, 47.6% (n=10) were unemployed, 38.1% (n=8) worked full time, and 14.3% (n=3) worked part time.

Validated questionnaire comparison (pre- vs post-intervention)

Over the course of the eight weeks, subject attendance ranged from 61-95%. Only 19 of the 21 participants filled out the post-intervention questionnaire. Thus, the pre-test results of the two participants that did not participate in the post-intervention questionnaire were excluded from the post-intervention results. For these results, a total of seven questions were used in addition to measuring weight change and BMI. An additional two questions were added to the post-intervention questionnaire where participants were allowed to add comments/suggestions they had for the program, and what changes they noticed in their cooking/eating habits.

A paired sample t-test showed that pre-intervention levels of confidence (mean 58.2, SD 9.6) were significantly lower than post-intervention levels (mean 70.68, SD 6.83) (p < 0.05). Refer to Table 2 for individual mean responses for each question pre- and post-intervention.

**Table 2:** Participants' confidence in different areas before and after intervention. Participants were given the option of 1 (not confident at all), 2 (not very confident), 3 (neither confident nor not confident), 4 (confident), or 5 (extremely confident).

	Pre-Intervention		Post-Intervention	
	Mean	Standard Deviation	Mean	Standard Deviation
Eating fruits/vegetables every meal	3.53	1.22	4.00	0.88
Eating fruits/vegetables as a snack	3.21	1.32	3.95	0.97
Eating 5 servings of fruits/vegetables each day	3.05	1.22	3.84	1.07
Cooking from basic ingredients	3.95	1.03	4.53	0.61
Knife skills in the kitchen.	3.95	1.27	4.58	0.51
Planning nutritious meals	3.63	1.17	4.58	0.61

A paired sample t-test of Enjoyment/Health behaviors which included questions such as if they enjoy cooking, if they have fresh fruit/vegetables in their refrigerator, and if cooking is a good use of their time, showed that pre-intervention levels (mean 8.57, SD 1.42) were significantly lower than post-intervention levels (mean 9.15, SD 0.89) (p < 0.05).

As this study was conducted online, participants self-reported weight at both baseline and after the eight classes had taken place. At baseline, mean weight was reported at 170.37 lbs ±33.23, while post-intervention, it decreased to 166.63 lbs ±31.68, which was a statistically significant mean weight change of -3.74 lbs (p=0.006). As for mean BMI, this was found to be 28.79±7.16 at baseline and 28.12±6.69 post-intervention. This also showed a statistically significant mean BMI change of -0.66 (p=0.004).

Participants’ cooking and culinary beliefs also showed notable changes. After participating in the intervention, 18 participants found cooking to be a good use of their time and even found enjoyment in cooking. This was also observed through the post-intervention participant feedback. These participants also shared that they have been cooking more at home and eating out less after partaking in Recipes For Health.

## Discussion

The results of this study show exciting data that support this project’s objectives and aim that a healthy cooking intervention can increase nutrition knowledge, increase healthy food choices, increase fruit and vegetable consumption, increase food preparation skills, and improve weight and BMI in participants. 

In comparison to the baseline, participants showed significant improvement after completing the study in that they reported having prepared vegetables in the refrigerator to be used during mealtimes. During week two, participants were educated on the importance and benefits of meal prepping and were also provided with simple tips to make meal prepping easy. The Recipes for Health workbook also included a meal-planning guide and meal planner that participants could use from week to week. Therefore, this significant change could be associated with the education and tools provided.

The effect of the intervention significantly improved the levels of confidence in the participants. There was a remarkable improvement in the participant’s confidence in planning nutritious meals. Through better planning of meals, participants also reported increasing their confidence in eating the recommended five servings of fruits and vegetables. This could be related to the variety of nutrition education topics that were covered throughout the eight weeks. Some of the topics covered included MyPlate, meal planning, mindful eating, and how to decrease saturated/trans-fat, sodium, and added sugar intake. In their comments, the participants reported that it was extremely helpful that the classes began with a nutrition education portion that was then reiterated through the recipes. The participant’s comments and answers to the questionnaire validate that a culinary class coupled with nutrition education can promote notable changes in dietary habits. 

Changes in dietary habits and eating behaviors were also seen in BMI and weight. After participating in the eight-week intervention, it was found that participants' BMI and weight significantly decreased. Obesity has been previously linked to a lack of practical meal preparation skills [5]. Individuals that are obese have an increased risk for cardiovascular disease, type 2 diabetes, renal disease, and cancer [2-3]. While our population was mostly overweight, our research highlights the potential of a culinary intervention to promote weight loss amongst its participants which may lead to a decreased chronic disease prevalence. 

From baseline to post-intervention, there was a notable increase in the number of participants that found it easy to prepare meals. It has been shown that eating out contributes to a higher BMI and its existing comorbidities and thus preparing most meals at home might be a way to help with weight management [7]. Our results support the statement of Alpaugh and colleagues that if people are equipped with proper cooking skills and nutrition-related knowledge they are more likely to cook at home; thus, improving diet quality and promoting healthy food intake [7]. 

Interventions that focus on teaching participants cooking strategies have been shown to be beneficial in the past. Reicks et al. conducted a systematic review of 28 research studies and found that cooking interventions led to positive changes in health status and improved intake of fiber, fat, and sodium, which have all been associated with the onset of chronic disease [9]. Adhering to a diet that follows the dietary guidelines is pivotal to decreasing overweight and obesity rates, and reducing the risk of chronic diseases such as cardiovascular disease and cancer [10]. The benefits of cooking at home go beyond the improvement of health status. Studies have found that individuals with greater cooking skills not only have better nutritional indicators but report better family connections, greater mental wellbeing, and lower levels of self-reported depression [11]. These psychosocial factors are important to note as they play a role in overall wellbeing and quality of life. Future studies could look into the impact a cooking intervention has on other elements of wellness such as mental health and family dynamics. 

Similar results were observed by Razavi et al., who observed that participants who followed a six-week hands-on, culinary nutrition education were nearly three times as likely to follow a Mediterranean dietary pattern and experience an increase in Mediterranean diet adherence [12]. Another study conducted by Metcalfe et al. showed no difference in the frequency of cooking but an increase in fruit and vegetable consumption in participants following a seven-week nutrition education and cooking class compared to a control group [13]. 

Throughout the eight weeks of our study, attendance remained above 60%, and no participant dropped out. This could be attributed to the fact that the participants were very interested and motivated to participate to help manage their chronic condition. In addition, participants were incentivized with a $15 gift card that they would receive after each class to account for purchasing groceries for the class. It also helped that the classes were conducted virtually ensuring that participants could attend from their homes. Many participants shared that they benefited from the classes including a nutrition-education portion followed by a cooking class. This allowed them to put into practice the information that they were being taught. Another strength is the fact that all recipes were budget-friendly and easy to modify according to each individual’s dietary preferences/needs. All the recipes were culturally diverse, including recipes from Mexico, Cuba, Italy, Jamaica, China, and Greece, among others. This allowed participants to get a taste of different cuisines from the comfort of their homes. 

Limitations to this study include that individuals of low socioeconomic status may not have joined due to no computer or smartphone access. Research by Garcia and colleagues revealed that cooking interventions are most beneficial to vulnerable individuals such as those of low-socioeconomic groups as it leads to increased food literacy [5]. Additional research reiterates that cooking classes allow low-income families to access healthier foods and increase food-preparation skills [14]. Low-income individuals have higher rates of chronic disease which leads to fair/poor health, activity limitations, and even a younger death when compared with those of higher incomes [15]. To allow for low-income individuals who may not have access to a computer or smartphone to participate, future studies could be conducted in person. 

In addition, since the study was completely virtual, we relied on self-reported weight and we were unable to obtain additional biometrics such as a lipid panel or a body composition analysis. Relying on self-reported weight and self-administered questionnaires may lead to bias, which can affect the accuracy of the results. Participation was also difficult to control given that some participants turned their cameras off during the sessions. It would be beneficial for future studies to be conducted in person to better assess participation. Future research should study how a culinary intervention coupled with nutrition education promotes change compared to participants that would solely receive nutrition education.

## Conclusions

The primary objectives of this study were met, which includes increasing nutrition knowledge/healthy food choices and improving food preparation skills. Our secondary objective to improve weight/BMI was also met. Despite the limitations of conducting this study virtually, the results of this study, as well as participant feedback showed positive outcomes. Further studies should test a larger sample size to include individuals of different backgrounds/socioeconomic status and measure the same outcomes six months post-intervention to ensure the sustainability of the results. In addition, future research should include a control group and be conducted in person to decrease bias and increase accuracy. All in all, with our preliminary data, this study shows that an online healthy cooking intervention can significantly improve BMI in individuals with chronic disease.
